# Case report: 7p22.3 deletion and 8q24.3 duplication in a patient with epilepsy and psychomotor delay—Does both possibly act to modulate a candidate gene region for the patient’s phenotype?

**DOI:** 10.3389/fgene.2022.1061539

**Published:** 2023-01-09

**Authors:** Rahma Touhami, Hajer Foddha, Eudeline Alix, Afef Jalloul, Soumaya Mougou-Zerelli, Ali Saad, Damien Sanlaville, Amel Haj Khelil

**Affiliations:** ^1^ Laboratory of human genome and multifactorial diseases, Faculty of Pharmacy, University of Monastir, Monastir, Tunisia; ^2^ Department of Cellular and Molecular Biology, Superior Institute of Biotechnology, University of Monastir, Monastir, Tunisia; ^3^ Laboratory of Cytogenetics, Hôpital Mère-enfant, CHU Lyon, Lyon, France; ^4^ Laboratory of Cytogenetics, molecular genetics, and human reproduction biology, CHU Farhat Hached, Sousse, Tunisia

**Keywords:** Psychomotor delay, epilepsy, facial dysmorphism, array CGH, deletion/duplication, BRAT1 gene

## Abstract

**Background:** Psychomotor delay, epilepsy and dysmorphic features are clinical signs which are described in multiple syndromes due to chromosomal imbalances or mutations involving key genes implicated in the stages of Early Embryonic Development. In this context, we report a 10 years old Tunisian patient with these three signs. Our objective is to determine the cause of developmental, behavioral and facial abnormalities in this patient.

**Methods:** We used banding cytogenetics (karyotype) and Array Comparative Genomic Hybridization (Array CGH) to this purpose.

**Results:** The karyotype was in favor of a derivative of chromosome 7 in the patient and Array CGH analysis revealed a loss of genetic material in 7p22.3-p22.1 (4,56 Mb) with a gain at 8q24.23-q24 (9.20 Mb) resulting from maternal 7/8 reciprocal translocation. An *in silico* analysis of the unbalanced region was carried out and showed that the 7p22.3-p22.1 deletion contains eight genes. Among them, *BRAT1* gene, previously described in several neurodevelopmental diseases, may be a candidate gene which absence could be correlated to the patient’s phenotype. However, the 8q24.23-q24 duplication could be involved in the phenotype of this patient.

**Conclusion:** In this study, we report for the first time a 7p deletion/8q duplication in a patient with psychomoteur delay, epilepsy and facial dysmorphism. Our study showed that Array CGH still useful for delivering a conclusive genetic diagnosis for patients having neurodevelopmental abnormalities in the era of next-generation sequencing.

## Introduction

Neurodevelopmental disorder with psychomotor delay, whether or not associated with intellectual disability or dysmorphism, show a complex etiology. Some of them are due to single gene mutations under autosomal dominant (Joshua et al., 2016), recessive (Rossi et al., 2017) or X-linked (Brand et al., 2021) genetic transmission. Others are syndromic and most often involve chromosomal aberrations in number or structure. Copy number variations (CNV) present important source of genomic variation in the general population, but are also well-known causes of many neurodevelopmental disorders (Shoukier et al., 2013) and could deregulate gene function *via* several different mechanism.

Among the reported patients with epilepsy and intellectual disability, about 6% have chromosomal abnormalities and to date more than 400 different chromosomal imbalances have been associated with seizures consisting in either deletions or duplications (Singh et al., 2020).

Micro-deletions and duplications in certain chromosomes have been described in neurodegenerative diseases. The development of molecular cytogenetics like FISH (Fluorescence *in situ* hybridization) and Array CGH Comparative Genome Hybridization (Vissers et al., 2003) have made it possible to tackle the diagnostic difficulties for this type of diseases. *In vitro* and *in vivo* functional tests based on knockout or site-directed mutagenesis methods allow confirmation of the involvement of deleted genes in the pathology (Alberts et al., 2002).

In this context, we report the case of a patient with psychomotor developmental delay, status epilepticus and facial dysmorphism for whom we found a 4.56 Mb deletion and 9.20 Mb duplication on chromosomes 7 and 8 respectively.

### Patient information

• De-identified patient specific information.

The study concerns a 10 years boy patient presented at the clinical exploration, in the Cytogenetic consultation at Farhat Hached Hospital of Sousse.

Written informed consent to participate to the study and to publish the results was obtained from the patient’s parents.

• Primary concerns and symptoms of the patient.

The patient presented a psychomotor developmental delay, status epilepticus and facial dysmorphism.

• Medical, family, and psychosocial history including relevant genetic information.

Medical information’s: 1) psychomotor delay (walking at the age of 2 years, holding the head at 8 months; sitting position at 1 year), 2) status epilepticus (all four limbs hypertonia with revulsion of the eyeballs) starting at 3 years of age, tonic-clonic epilepsy at the age of 3 years with revulsion of the eyeballs and fever at 38.5°C, 3) facial dysmorphism.

Family and psychosocial history: the patient was born from consanguineous parents, by Cesarean delivery without perinatal pain and childbirth complication. No family member shows the patient’s signs.

• Relevant past interventions and their outcomes.

On finding epilepsy, the patient was put on Depakine at 3 years, stopped by the mother after 6 months, what spawned the recurrence of tonic-clonic seizures at the age of 4.5 years. Finally, he was put back on Depakine without recurrence of seizures.

### Clinical findings

At 10 years, the patient presents a delay of language relating to the two sides expressive and receptive, a reflex quadripyramidal syndrome, a dysmorphic syndrome.

### Timeline

Relevant data from the episode of care are showcased in Table 1.

**TABLE 1:** Relevant data from the episode of care.

Psychomotor development	Age
Headwear	8 months
Sitting position	1 year
Walk	2 years
Language	Absent

Progression of the disease	
Status epilepticus with 38.5 °C fever	3 years
Recurrence of tonic-clonic epilepsy	4.5 years

Treatment	
Depakine	3 years
Stop Depakine	3,5 years
Put back on Depakine	4,5 years

### Diagnostic assessment

#### Diagnostic methods

Physical Examination (PE): weight 16 Kg, height 110 cm, cranial circumference: 49 cm, presents a good contact. Examination of facial dysmorphism showed broad forehead, facial clefts, wide nose base, hypertelorism, slits oblique palpebrals below and outside, short philtrum.

Magnetic Resonance Imaging (MRI): bilateral and symmetrical T2 hypersignal involving the parieto-occipital and bilateral frontal periventricular white matter as well as the two semi-oval centers respecting the gray matter in places and affecting the U-shaped fibers in places, evoking leukodystrophy.

Electro-encephalogram (EEG): poor organization of waking and sleeping EEG activity with absence of paroxysmal abnormalities.

Sleep EEG: paroxysmal generalized interictal abnormality.

Electroneuromyogram (ENMG): without anomaly.

Bit error ratio (BER): Normal, hearing threshold at 30 dB on both sides.

Eye examination: normal.

Biological balance: normal.

• Diagnostic challenges.

Search for genetic abnormalities of a possible syndromic leukodystrophy.

• Prognostic characteristics

Not applicable.

• Diagnosis (including other diagnoses considered).

Syndromic leukodystrophy was suggested given the facial dysmorphism. This suggestion has to be confirmed by genetic explorations which began by banding cytogenetics followed by molecular cytogenetic techniques.

#### Banding cytogenetics

**FIGURE 1:**
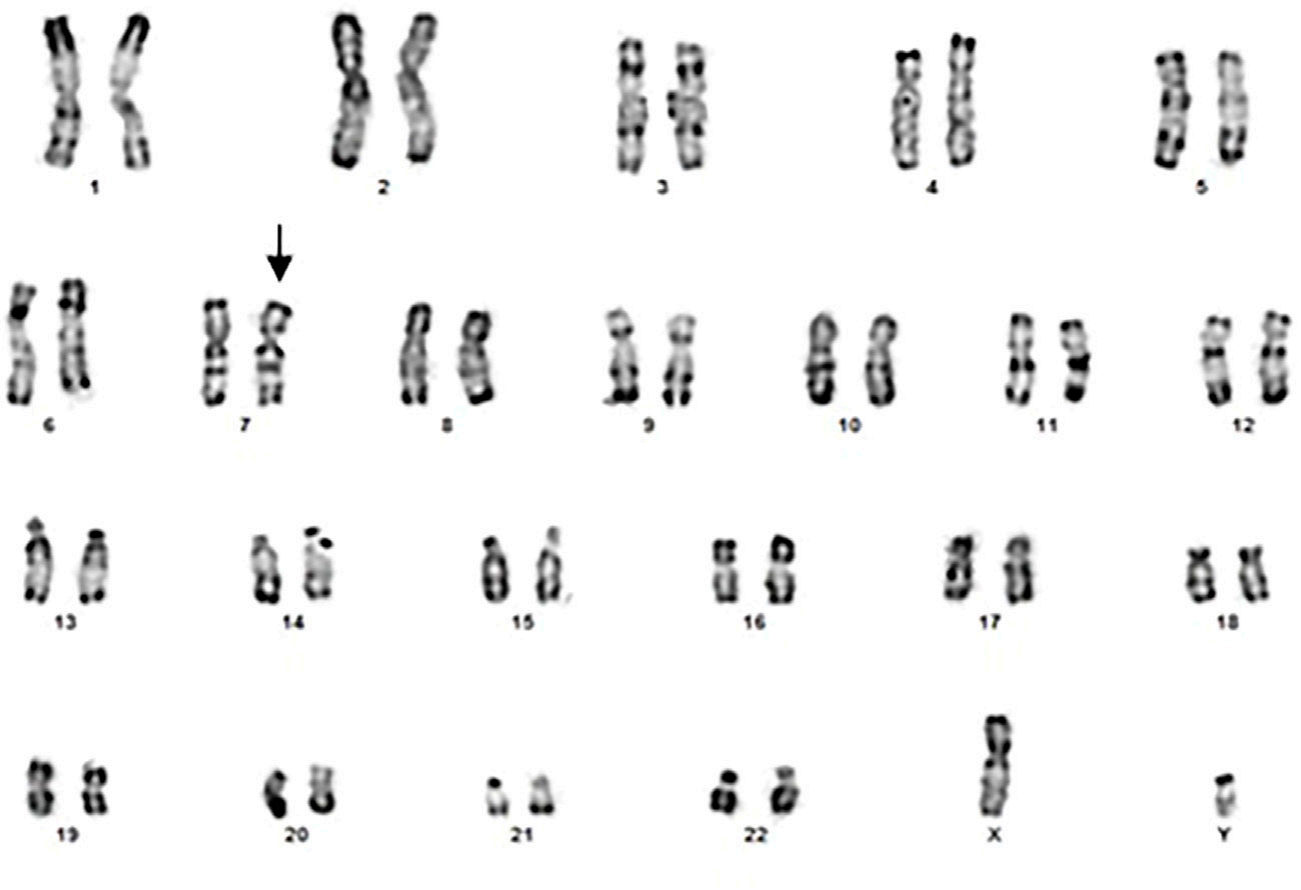
Banding cytogenetics of the patient in favor of a derivative of chromosome 7 (arrow).

Using R banding, it appears normal (46, XY) but it could present a derivative of chromosome 7 (Figure 1) which remains to be confirmed by more efficient methods.

**FIGURE 2:**
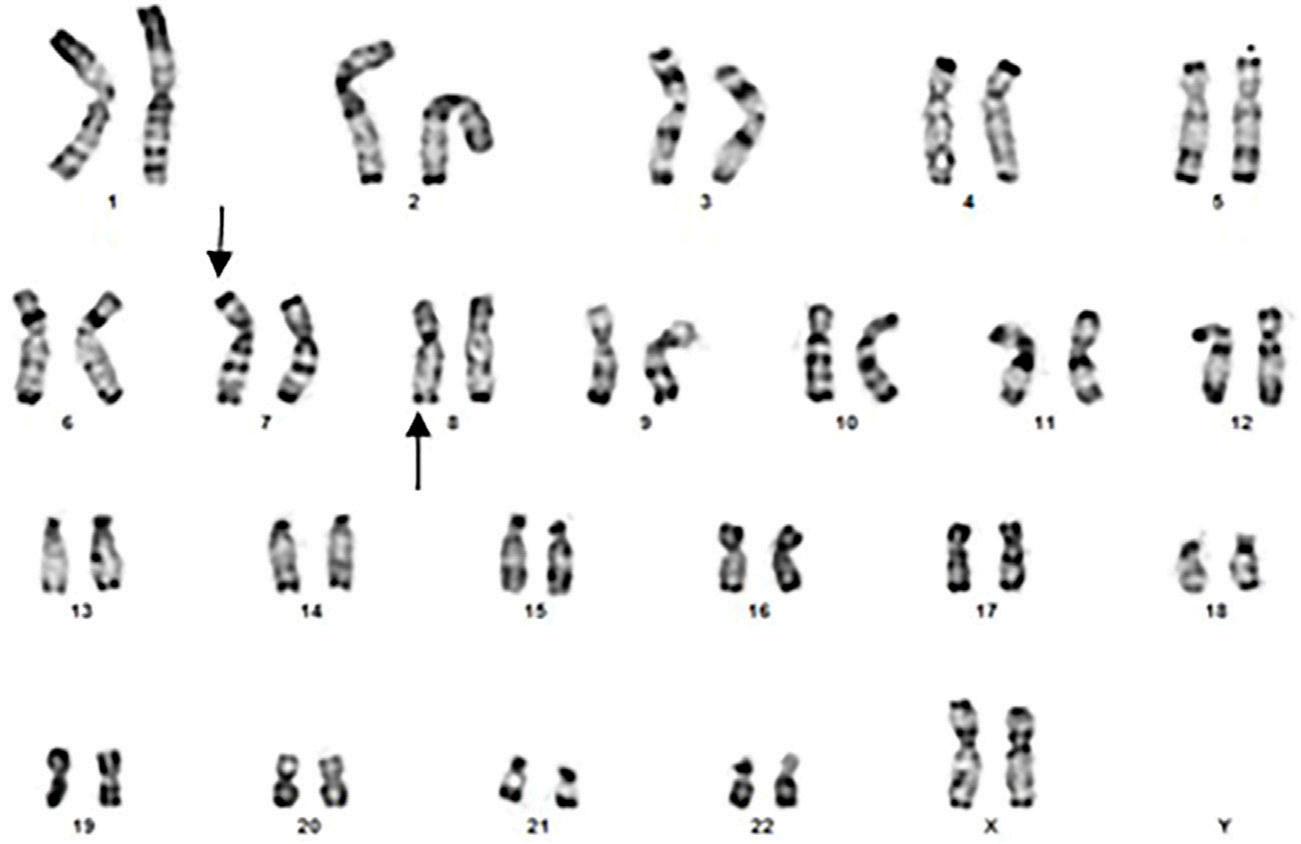
Banding cytogenetics of the mother showing a karyotype in favor of 7/8 reciprocal translocation (arroaws).

The cytogenetic exploration of the parents showed a normal karyotype for the father and a reciprocal translocation between chromosomes 7 and 8 in the mother (Figure 2), which karyotype formula is: 46,XX.t (7; 8) (p22.3-p22.1; q24.23-q24.3). The patient was born from an adjacent 1 maternal gamete carrying a normal chromosome 8 and a derivative of chromosome 7.

#### Molecular cytogenetics

##### Method

An Array CGH using Agilent plateform with 4 × 180K resolution was performed for this patient in the Cytogenetic Service of CHU of Lyon, France. The 180k slides were scannned on agilent DNA microarray scanner and images were extracted with feature extraction software 12.0.1.1. Data analysis was carried out with cytogenomics v3.0.3.3. Data interpretation analysis used workbench v3.4.2.7.

##### Results

**FIGURE 3:**
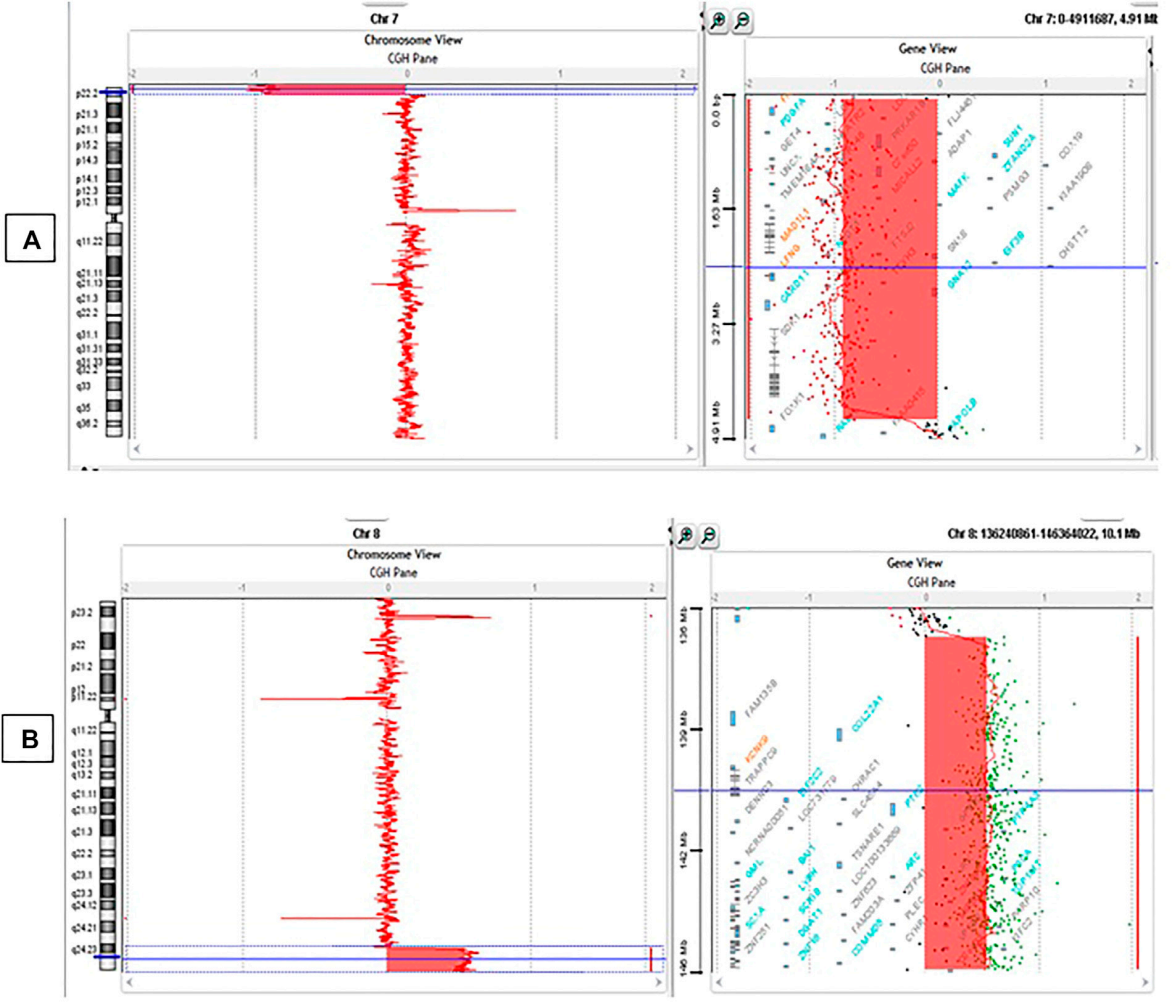
Array CGH results. **(A)** Deletion of 4.56 Mb in 7p22.3-p22.1 ranging from 83325bp to 4642192bp (hg19); **(B)** Duplication of 9.20 Mb in 8q24.23-q24.3 ranging from 137078730bp to 146280020bp (hg19).

Data analyses of the Array CGH confirmed the karyotype result: Partial deletion of 7p chromosome and partial duplication of 8q chromosome were observed (Figure 3). According to the new ISCN 2020 (International System for Human Cytogenomic Nomenclature) changed in 2016 (Liehr, 2021), the formula in this patient was:

46,XY,der(7)t(7; 8)(p22.3; q24.3).arr[GRCh37] 7p22.3-p22.1(83325_4642192)x1, 8q24.3(137078730_146280020)x3.

##### Discussion

###### The 4.56 Mb 7p22.3-p22.1 deletion

To our knowledge, this is the first report of a patient with 4.56 Mbp deletion on chromosome 7p22.3-p22.1 inherited from a maternal reciprocal translocation.

In the literature, large genetic deletions or duplications have been associated with Neurodevelopment disorders. Singh et al. have described a 1.6 Mbp deletion on chromosome 3q29, including 21 genes which deletion may increase apoptosis that disrupts cellular organization and brain morphology (Singh et al., 2020).

In our patient, the analysis of the deleted region on chromosome 7p22.3-p22.1 using ALAMUT software showed that this 4.56 Mbp deletion encompasses eight genes: *INT1, FAM20C, LFNG, MAD1L1, DNAAF5, BRAT1, IQCE, CARD11*, and *AP5Z1*. Bioinformatic functional analysis showed that of these genes, only *BRAT1* (BRCA1-associated ATM activator 1) was related to the patient’s phenotype. The *BRAT1* gene enables protein binding and is involved in numerous cellular functions such as apoptotic process, cell migration and proliferation, cellular response to DNA damage stimuli, cell growth and regulation of protein phosphorylation (Aglipay et al., 2006; So and Ouchi, 2013). Recently, BRAT1 deletion is described to disrupt the functions of Integrator complex that processes 3′ ends of various non-coding RNAs and pre-mRNAs. In particular, defects in BRAT1 impede proper 3′ end processing of UsnRNAs and snoRNAs, replication-dependent histone pre-mRNA processing, and alter the expression of protein-coding genes (Cihlarova et al., 2022).

BRAT1 gene is described to be implicated in several diseases and syndromes. Homozygous and compound heterozygous mutations in the *BRAT1* gene were identified in patients with Neurodevelopmental disorder with cerebellar atrophy and with or without seizures (Saitsu et al, 2014; Hanes et al., 2015; Mundy et al., 2016; Srivastava et al., 2016; Mahjoub et al., 2019; Nuovo et al., 2022). Recessive homozygous and compound heterozygous *BRAT1* mutations are also described to be a cause of epilepsy of infancy with migrating focal seizures (Scheffer et al., 2020) and Lethal Neonatal Rigidity and Multifocal Seizure Syndrome (Hanes et al., 2015; Celik et al., 2017; Van Ommeren et al., 2018; Balasundaram et al., 2021; Li et al., 2021). Some authors expanded the phenotypic spectrum of *BRAT1* related disorders by reporting on patients with various *BRAT1* mutations resulting in clinical severity that is either mild or moderate compared to the severe phenotype seen in RMFSL (Rigidity and Multifocal Seizure Lethal) (Mundy et al., 2015; Fernández-Jaén et al., 2016; Srivastava et al., 2016). An intronic variant in *BRAT1* creating a cryptic splice site has been described to cause epileptic encephalopathy without prominent rigidity (Colak et al., 2020).

Our patient exhibits, with patients described in the above cited literature and having *BRAT1* abnormalities some clinical similarities including psychomotor developmental delay, status epilepticus, hypertonia and facial dysmorphism. Based on these similarities, we suggest that the absence of the entire *BRAT1* gene on one of the two 7 chromosomes due to the 7p22.3-p22.1 deletion may be responsible for the clinical features of our patient.

##### Phenotype/genotype correlation

The *BRAT1* gene encodes the BRCA1-associated protein required for ATM activation-1, a protein that interacts with BRCA1 and ATM to initiate DNA repair in response to DNA damage (Balasundaram et al., 2021). BRAT1 functions as an activator of ATM into the cell nucleus by maintaining its phosphorylated status while also keeping other phosphatases at bay (Low et al., 2015).

BRAT1 also acts as a regulator of cellular proliferation and migration and is required for mitochondrial function. Disruption of BRAT1 function in RMFSL has been proposed to cause dysfunction in the DNA damage response pathway and impair mitochondrial homeostasis (Van Ommeren et al., 2018). In addition, functional studies revealed that loss of *BRAT1* expression significantly decreases cell proliferation and tumorigenecity, remarkably lowers cell migration, and, interestingly, highly increases glucose uptake and production of mitochondrial reactive oxygen species (So and Ouchi, 2014). This dysfunction of DNA repair and the disruption of mitochondrial function may be related to the psychomotor developmental delay and facial dysmorphism observed in our patient. When occurring in the brain, these dysfunctions may explain the focal interictal paroxysmal abnormalities (Mariani et al., 2011) and the leucodystrophy leading to the status epilepticus.

However, we could not exclude the supplementary action of the contiguous deleted genes at 7p22.3-p22.1 and the duplicated ones at 8q24.2, previously reported as associated to epilepsy.

###### The 9.20 Mb duplication on 8q24.23-q24.3

Duplications on several chromosomes are described in some cases of psychomotor development. For example, a 17q21.31 microduplication, was described in a girl with severe psychomotor developmental delay and dysmorphic craniofacial features (Kirshhofet al, 2007), and a 12.4 Mb duplication of 17q11.2q12 was reported in a patient with psychomotor developmental delay and minor anomalies (Casellia et al., 2010). In addition, a literature review described Xq13.3-q21.1 duplication in males with syndromic intellectual disability and congenital abnormalities (Chen et al., 2017).

The 9.20 Mb duplicated region found in our patient on chromosome 8 (8q24.23-q24.3) (from 137078730 to 146,280,020 bp) contains numerous genes. Among them, *KCNK9* gene showed mutations reported in maternally inherited Birk Barel syndrome (Barel et al., 2008), and the *TRAPPC9* gene was described in non-syndromic familial forms of intellectual deficiency (Mir et al., 2009). In addition, *KCNQ3* gene was related to disorders including benign familial neonatal epilepsy and benign familial infantile epilepsy, seizure disorders that occur in children who typically have normal psychomotor development (Miceli et al., 2014; Springer et al., 2021) and autism (Sands et al., 2019), and *GRINA* gene described in Central Nervous System Diseases (Chen et al., 2020).

Our patient doesn’t show any sign of intellectual deficiency, supporting the hypothesis that, in this case reported, KCNK9 and TRAPPC9 should not be implicated. However, we cannot exclude the hypothesis of the implication of the 8q duplication in the pathogenesis of this patient even though the 7p22.3-p22.1 deletion is more likely to be related to the phenotype.

### Therapeutic intervention

The treatment is essentially based on Depakine (200-0-200/d) with motor and speech therapy rehabilitation.

### Follow-up and outcomes

Medical treatment has prevented relapses of epileptic seizures. Motor and speech rehabilitation gave a slight improvement. Finally, the discovery of this chromosomal anomaly and its association with the patient’s phenotype makes it possible to give good genetic advice to the family considered and to plan a prenatal diagnosis for subsequent pregnancies in the family carrying this anomaly.

## Conclusion

We report here, using array Comparative Genomic Hybridization, a novel deletion on 7p22.3-p22.1 in a Tunisian patient with psychomotor developmental delay, status epilepticus and facial dysmorphism. The loss of *BRAT1* gene which is included in the deletion may be related to the patient’s phenotype. This work highlights the interest of array CGH to further characterize the karyotype results for neurodegenerative and psychomotor diseases diagnosis. The association of leukodystrophy with facial dysmorrhia and psychomotor retardation could constitute syndromic leukodystrophy secondary to deletion 7p. However, in our case, further investigations should be done to confirm the *BRAT1* gene implication as genetic etiology and to get a better understanding of the molecular and cellular mechanisms that lead to this disorder.

## Data Availability

The datasets for this article are not publicly available due to concerns regarding participant/patient anonymity. Requests to access the datasets should be directed to the corresponding author.
